# Foreign Accent Syndrome As a Psychogenic Disorder: A Review

**DOI:** 10.3389/fnhum.2016.00168

**Published:** 2016-04-27

**Authors:** Stefanie Keulen, Jo Verhoeven, Elke De Witte, Louis De Page, Roelien Bastiaanse, Peter Mariën

**Affiliations:** ^1^Department of Linguistics and Literary Studies, Clinical and Experimental Neurolinguistics, Vrije Universiteit BrusselBrussels, Belgium; ^2^Department of Linguistics, Center for Language and Cognition, Rijksuniversiteit GroningenGroningen, Netherlands; ^3^Department of Language and Communication Science, School of Health Sciences, City University LondonLondon, UK; ^4^Department of Linguistics, Computational Linguistics and Psycholinguistics Research Center, Universiteit AntwerpenAntwerp, Belgium; ^5^Department of Psychology, Clinical and Lifespan Psychology, Vrije Universiteit BrusselBrussels, Belgium; ^6^Department of Neurology and Memory Clinic, ZNA Middelheim General HospitalAntwerp, Belgium

**Keywords:** foreign accent syndrome, psychogenic, non-organic FAS, speech disorder, review

## Abstract

In the majority of cases published between 1907 and 2014, FAS is due to a neurogenic etiology. Only a few reports about FAS with an assumed psychogenic origin have been published. The present article discusses the findings of a careful database search on psychogenic FAS. This review may be particularly relevant as it is the first to analyze the salient features of psychogenic FAS cases to date. This article hopes to pave the way for the view that psychogenic FAS is a cognate of neurogenic FAS. It is felt that this variant of FAS may have been underreported, as most of the psychogenic cases have been published after the turn of the century. This review may improve the diagnosis of the syndrome in clinical practice and highlights the importance of recognizing psychogenic FAS as an independent taxonomic entity.

## Introduction

It has now been over a century that researchers have reported on a motor speech disorder most frequently referred to as “Foreign Accent Syndrome” (FAS). The first patient with FAS was anecdotally described by Marie (1907). The term “FAS” was later coined by Whitaker (1982) who also proposed a set of diagnostic criteria: (1) “the accent is considered by the patient, by acquaintances and by the investigator, to sound foreign”; (2) “it is unlike the patient's native dialect before cerebral insult,” (3) “it is clearly related to central nervous system damage (as opposed to an hysteric reaction, if such exist)”; (4) “(t)here is no evidence in the patient's background of being a speaker of a foreign language (i.e., this is not like cases of polyglot aphasia)” (Whitaker, 1982, pp. 196 and 198). These criteria only apply to one of the three FAS subtypes in the taxonomic classification recently developed by Verhoeven and Mariën (2010), who distinguished between a neurogenic (including a developmental subtype), a psychogenic and a mixed variant of FAS.

Psychogenic FAS is defined by Verhoeven and Mariën (2010) as “the variant in which the foreign accent of the patient is grounded in underlying psychological issues” (p. 601). It is also referred to as “non-organic,” “functional,” or “psychosomatic” FAS. Aronson and Bless (1990) have expressed a clear preference for the term “psychogenic” because this term has “the advantage of stating positively, based on an exploration of its causes, that the […] disorder is a manifestation of psychological disequilibrium such as anxiety, depression, personality disorder, or conversion reaction […]” (p. 121). In general, this “sub-category” contains all the cases of FAS in which an organic substrate cannot be identified after careful clinical neurological, neuroradiological, and/or neurophysiological examination, and for which a clear psychological factor is identified (e.g., Verhoeven et al., 2005) as well as the cases for which it is hypothesized that a disclosed organic deficiency cannot be held responsible for the FAS (e.g., Gurd et al., 2001; Van Borsel et al., 2005). The latter is not uncommon.

According to Baumgartner (1999) several researchers in speech and language pathology have published cases in which a clear neurological impairment was identified, but the speech or voice disorder was convincingly argued to be of psychogenic origin (Tippett and Siebens, 1991; Baumgartner and Duffy, 1997). Baumgartner (1999) emphasizes the importance of carefully considering the patient's medical history, meticulously interpreting the symptoms, and evaluating the coherence between different observations. If medical history, onset of symptoms, symptom characteristics and their evolution, neurological examinations, neuroimaging, and cognitive work-up do not unambiguously point toward a neurological disorder, an alternative interpretation should be considered.

This article presents a detailed review of FAS cases with an assumed psychogenic etiology published between 1907 and July 2014. The focus of the investigation is on the associated psychopathologies, the onset and remission of the accent, the type of accent, the segmental, and suprasegmental characteristics contributing to the perception of the patient's accent as “foreign,” as well as the comorbid speech- and/or language symptoms.

The goal of this review is to analyze the main features of psychogenic FAS in order to shed more light on this taxonomic variant and facilitate the diagnosis in clinical practice.

## Methods

The available literature on (psychogenic) FAS was identified by means of regular searches in online electronic databases (*Web of Knowledge, ScienceDirect, PubMed, Medline, PsycINFO*), using the following keywords in Boolean search: “foreign accent syndrome,” “FAS,” “psychogenic AND FAS,” “psychogenic AND foreign accent syndrome.” The reference sections of all relevant articles were scanned to identify additional references. All the articles between 1907 and July 2014 were included. Only original case descriptions were retained for this review, as some of the data were re-used by the same or other authors in later publications. Inclusion criteria for psychogenic FAS were: (1) the onset of a foreign accent, (2) the presence of, or indication(s) for psychological/psychiatric symptoms, (3) the absence of neurological damage that could explain the speech and/or language symptomatology

## Results

### Demographic characteristics and associated psychopathologies

The initial database search resulted in a corpus of 129 articles reporting instances of FAS (regardless of the etiology). However, at least 24 cases were published twice or more. Only original case reports were included for the counts in this section. Fifteen of the 105 (original) FAS cases published between 1907 and July 2014 matched the inclusion criteria of psychogenic FAS (see Table 1). The putative psychogenic FAS cases represent 14% of all published FAS cases (*n* = 15/105). Two case reports [case 3, 8] were reported twice^1^. Sixty-seven percent of the included patients are women (*n* = 10/15), and 33% are men (*n* = 5/15). The mean age of patients with assumed psychogenic FAS is 48 years and 1 month (range: 30–74 years, SD: 12 years and 9 months). Men had a mean age of 56 years and 2 months (range 30–74 years, SD: 17 years 8 months) and women 44 years and 1 month (range 32–54 years, SD: 7 years 11 months). Patient's occupation was only mentioned in a few case reports (*n* = 5/15) [cases 3, 5, 8, 10, 12]. Education levels were never stated. Five patients are described as right-handed [cases 2, 5, 8, 11, 12]. However, handedness was only formally assessed in one case (case 5: right-handed; Edinburgh Handedness Test; Oldfield, 1971). For the remaining cases [1, 3, 4, 7, 8, 10, 13–15], handedness was not indicated. Two patients were self-proclaimed monolinguals [cases 8, 9], whereas two were definitely polyglots [case 5: Dutch-French-English, case 10: English-Spanish]. In case 5, FAS affected both Dutch and English, but French was perfect on all linguistic levels (suprasegmental, segmental, morphology, syntax). In case 10, however, it was not mentioned to what extent the patient's proficiency of Spanish was affected. As far as the psychological disorder is concerned, 33% of the cases presented with conversion disorder (*n* = 5/15; cases 5, 9–12), 13% with schizophrenia (*n* = 2/15) [cases 3, 6], 13% with bipolar disorder (*n* = 2/15) [cases 7, 8], 13% with obsessive-compulsive disorder (OCD) (*n* = 2/15) [cases 14, 15], 7% with post-traumatic neurosis (*n* = 1/15) [case 1], and 7% with mania (*n* = 1/15) [case 13]. In 13% of the cases, no clear psychological disorder was associated with the FAS (*n* = 2/15) [cases 2, 4] (see Table 1). However, for these cases neurological and neurophysiological examinations as well as neuroimaging were regarded incompatible with a neurogenic etiology, and it was concluded that the FAS had to be non-organic in nature.

**Table 1:** **Overview of the psychogenic case reports (literature review: 1907- July 2014)**.

	**Case**	**Age/Gender/Handedness**	**Medical history**	**Neurological, biological, physical and/or radiological examination(s)**	**Psychological/psychiatric affectation**	**Accent**	**Comorbid speech and language disorders/symptoms**
1	Critchley, 1962 (Case 1) = Critchley, 1970 (Case 1)	49/F/NI	/	/	Post-traumatic neurosis after head injury	English 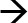 Welsh	/
2	Gurd et al., 2001	47/F/R	/	Nov. 1999: normal Doppler, normal MRI, CT: small high signal lesion in cerebellar vermis; Dec. 1999: tone, power, coordination, and reflexes in arms and legs were normal, gait disorder; MRI: several small foci of T2 hyperintensities in peripheral white matter of both frontal lobes, left inf. frontal corona radiata and left thalamus, EEG: sharp and slow waves, but no history of epilepsy; presence of oligoclonal bands in CSF	MS (?)	English (North Yorkshire) 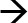 French	/
3	Reeves and Norton, 2001 = case 3 Reeves et al., 2007	65/M/NI	Psychotic exacerbations since thirties, schizophrenia at forty, Parkinson's disease with tremor in bilateral upper extremities, hypertension	MRI scan (with contrast): normal, Blood and histological exam: normal	(Positive) schizophrenia	American English 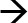 British English	/
4	Van Borsel et al., 2005	32/F/NI	Permanent right-sided neurosensory hearing loss with sloping configuration (as of the age of 6); age 23: head trauma and whiplash injury 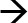 chronic headache; age 32: minor head trauma 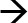 hoarseness 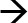 ENT exam was normal; onset of speech problems shortly of visit to ORL; on-going psychiatric history: depression (suicidal ideation); family problems.	No motor or sensory abnormalities; coordination, gait and posture: normal; CT: normal	Psychological impact, family problems + suicidal ideation	Dutch 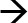 “awkward” accent	Mute (initially), agrammatism
5	Verhoeven et al., 2005 = Verhoeven et al., 2013, case 3	51/F/R formal test, polyglot	Disrupted speech and gait problems since 1995; wheelchair-bound; no history of developmental or psychiatric disorders	Two months after “near-accident” (1995): CT: normal; EEG: normal Repeat investigation in 2003: Gait: unsteady, wide-based, coordination, muscle tone and tendon reflexes: normal; CT and (struct.); MRI: normal; EEG; normal; laboratory studies, lumbar punction: normal	Psychotrauma 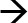 conversion disorder 1995: conversion disorder (MMPI) 2003: DIS-Q & MMPI: near normal	Dutch (The Netherlands) 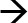 French	Paragrammatism?
6	Reeves et al., 2007, Case 1	30/M/NI	10-year history of schizophrenia,	Laboratory work-up, physical examination: normal; MRI scan: normal; EEG: normal; Blood exam: normal; SPECT: normal	Positive schizophrenia	Southern American English accent 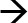 Jamaican accent	/
7	Reeves et al., 2007, Case 2	53/F/NI	30-year history of bipolar disorder	Laboratory work-up, physical examination: all normal; MRI scan: normal; EEG: normal; Blood exam: normal; SPECT: normal	Psychosis (bipolar disorder)	American English 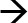 “European”	/
8	Poulin et al., 2007 = Roy et al., 2012	74/M/R	Epilepsy between 6 and 14 years; Bipolar disease as of 1982, multiple exacerbations; FAS first mentioned in 2003; Delirium due to lithium intoxication 6 months before FAS started; Tremor; Neurosensory hypoacusia	Neurological examination: Coordination and gait: decomposition of the half-turn, slight incoordination of left arm, micrographia; Primitive reflexes: palmomental and snout reflexes present; Radiological examination: MRI (Dec. 2005): normal, though slight atrophy in left sylvian fissure; 18-FDG PET scan: diffuse hypometabolism in frontal, parietal and temporal lobes and focal deficit concerning esp. the left sylvian sulcus	Bipolar disorder; recurrent psychotic episodes with manic exacerbations	Québec French 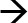 Acadian French/French of France/English	Mild agrammatism (as of 2002/2003), surface agraphia, Spanish and German sounding words come to mind: not able to suppress
9	Tsuruga et al., 2008	44/F/NI	End-thirties: nausea, vomiting, diarrhea, tinnitus, tired eyes, irritations: diagnosed with autonomic imbalance; Few years later: respiratory paroxysm, experienced aphonia (few hours) (hospitalized several), after violent familial experience: aphonia (2 days), loss of appetite, Later: FAS	Laboratory work-up: liver and thyroid: mild, though undefined abnormalities; MRI, SPECT, and EEG: normal	Conversion Disorder	Japanese 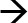 Chinese	/
10	Haley et al., 2010	36/F/NI polyglot: late bilingual (Spanish)	Admission**:** gait: unsteady; posture: left-sided weakness, sensory: visual blurring, altered hearing left ear, slurred speech, weakness of left side of the face, subtle weakness of left arm and leg. 10 days after symptom onset: speech impairment, trouble swallowing and abnormal sensations in the left face, arm, and leg. 5 days later (stroke specialist): symptoms worsened, FAS was established	MRI: normal, Echocardiogram: moderate mitral regurgitation (also 2 years prior), Blood analysis: normal. Impression of Bell's palsy, with additional conversion disorder symptoms. Follow-up**:** MRI (10 days later): no abnormalities, MR angiogram: no abnormality of the brain vasculature, CSF: no MS. Over subsequent months**:** several relapses, discontinuous periods with less accented speech, another brain MRI and cervical MRI during relapse: normal	Conversion disorder	English 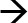 French, Spanish, Jamaican, Caribbean, African	/
11	Cottingham and Boone, 2010	36/F/R	Several hospitalizations for symptoms not explicable by neurological cause (e.g., sudden hoarseness of voice)	Motor vehicle accident, CT: (head): normal. Headaches 3 days after accident, facial numbness, weakness in right arm, speech difficulties: 10 days after accident. Later: deafness to left ear. Approx. 10 days post-onset: EEG, Brain MRA, MRI: normal, neurological examination: normal, but: speech apraxia + left-sided give-way weakness (non-neurological sign), dysarthria	Minor TBI/Conversion Disorder (?)	English 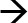 Eastern European accent (3 years after accident)	Initially dysarthric- or speech apraxic-like symptoms, telegraphic speech
12	Jones et al., 2011	39/F/R	Unremarkable	One month after symptom onset: sensory loss, effort-dependent inconsistencies in strength when testing extremities, gait: disturbed, fluctuations, uneconomic postures, dramatic give way weakness; positive “chair test”; speech: disrupted articulation and prosody; CT, MRI (brain + cervical), EEG: normal	Conversion Disorder	American English 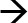 Jamaican accent	Initially mute
13	Lewis et al., 2012	54/F/NI	Unremarkable	CT (brain): normal	Mania	American English 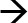 Caribbean English	/
14	Polak et al., 2013, case 1	47/M/NI	Refractory OCD for over 25 years	March 2006: 2 DBS electrodes 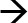 treatment; Pre-operative MRI and post-operative CT: no lesions	Refractory OCD (for >25 years)	Standard Dutch 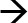 Pronounced regional Dutch accent	/
15	Polak et al., 2013, case 2	65/M/NI	Refractory OCD for over 50 years	/	Refractory OCD	Regional Dutch variant 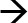 more sophisticated/formal Dutch	/

Relevant information (from left to right) includes the age, gender and handedness of the patients, their medical history, the neurological and neuroradiological exams, the psychological or psychiatric affectation, the accent, and the comorbid speech and language disorders.

### Phonetic characteristics

Neurogenic FAS has been associated with a very diverse set of segmental and suprasegmental pronunciation characteristics, often with great inter-patient variability. While some studies primarily investigated the phonetic and acoustic characteristics of FAS, others focused on the pathophysiological substrate of the syndrome (see also Ingram et al., 1992; Kanjee et al., 2010). This dissociation equally applies to psychogenic FAS: some researchers have focused on the identification of the associated psychopathology and the link between the psychological disorder and FAS (e.g., Reeves and Norton, 2001; Reeves et al., 2007), whereas others described the segmental and suprasegmental transformations in speech (Verhoeven et al., 2005; Haley et al., 2010). The speech characteristics are listed in Table 2.

**Table 2:** **Overview of the segmental and suprasegmental changes in the speech of assumed psychogenic FAS**.

**Segmental**	**Case numbers**	**Percentage (%) of psychogenic patients for whom speech characteristics were noted**
**CONSONANTS**		
Substitution (manner/place/aspect)	3, 4^*^, 5^*^, 8^*^, 9, 10^*^, 11, 12^*^, 13^*^	64
Omission	2, 4^*^, 6, 7, 9, 10^*^, 11, 13^*^	57
Addition	2, 4^*^, 5^*^	21
Cluster reduction	4^*^, 13	14
Increased friction	2	7
Lengthening	2	7
**VOWELS**		
Substitution	3, 4^*^, 5^*^, 12^*^, 13^*^	36
Lengthening	2, 3, 8^*^, 10^*^, 12^*^	36
Addition	5^*^, 11, 12^*^, 13^*^	29
Fronting	5^*^, 8^*^, 13^*^	21
Monophthongization of diphthongs	2, 10^*^, 12^*^	21
Reduced contrast	10^*^, 13^*^	14
Lenition	9, 10^*^	14
Backing	8^*^, 10^*^	14
Omission	12^*^	7
Shortening	9	7
Increased tenseness	10^*^	7
**Suprasegmental**	**Case numbers**	**Percentage (%) of psychogenic patients for whom speech characteristics were noted**
Abnormal intonation	3, 6, 7, 8^*^, 9, 10^*^, 11, 12^*^, 13^*^	64
Slow speech rate	5^*^, 8^*^, 10^*^, 11, 12^*^	36
Incorrect word stress	2, 4^*^, 5^*^, 10^*^, 11	36
Syllable-timed speech	2, 4^*^, 8^*^, 10^*^, 13^*^	36
Variable pitch	2, 10^*^, 12^*^	21
Hypernasality	10^*^, 11, 12^*^	21
Slow articulation rate (excluding pauses)	8^*^, 12^*^	14
Terminal pitch rise (errors)	7, 13^*^	14
Larger than normal F0 excursions	8^*^, 10^*^	14
Excessive pausing	5^*^, 13^*^	14
Fast speech rate	13^*^	7
Terminal pitch fall (errors)	8^*^	7

Cases marked by an asterisk are cases for which formal phonetic and acoustic analyses were carried out. For the remaining cases, the characteristics were noted based on perceptual (impressionistic) phonetic analysis.

All the speech characteristics in Table 2 have been reported for patients with neurogenic FAS as well. It seems that in patients considered as psychogenic, vowels are more often affected than consonants and this also seems to hold for neurogenic patients (Ingram et al., 1992; Miller et al., 2006; Katz et al., 2008; Van der Scheer et al., 2014). Moreover, the nature of the changes is different for vowels and consonants: consonants are mainly affected by substitutions, omissions and additions, whereas errors against vowels mostly consist of substitution errors, vowel lengthening, and additions.

### Accents associated with psychogenic FAS

Table 3 shows the variety of accents associated with psychogenic FAS.

**Table 3:** **Overview of the different accents associated with FAS**.

**Case**	**Pre-FAS accent**	**Newly developed accent**
Case 1	British English	Welsh
Case 2	British English (North Yorkshire)	French
Case 3	American English	British English
Case 4	Dutch (Belgium)	“An awkward accent”
Case 5	Dutch (The Netherlands)	French
Case 6	Southern American English	Jamaican English
Case 7	American English	“European”
Case 8	Montréal French	Acadian French, French of France, or English
Case 9	Japanese	Chinese
Case 10	American English	Eastern European
Case 11	English	French/Spanish/Jamaican/Caribbean/African
Case 12	American English	Jamaican English
Case 13	American English	Caribbean English
Case 14	Standard Dutch (The Netherlands)	Regional variant of Dutch (The Netherlands)
Case 15	Regional Dutch (The Netherlands)	Standard Dutch (The Netherlands)

In 9 out of 15 cases (60%) the accent changed between geographical variants of the same language [cases 1, 3, 6, 7, 11–15]. In 9 cases (60%) the mother tongue was a variant of English (either American or British, or a regional variant) [cases 1–3, 6, 7, 10–13]. In four cases, other variables, such as pathological language mixing [case 5] and code switching [cases 3, 14, 15], might have created the impression of FAS.

### Onset and remission of the accent

An acute onset of FAS occurred in 7 cases [cases 3, 6–8, 13–15]. In these cases, FAS was associated with mania [case 13], bipolar disorder [cases 7, 8], and obsessive-compulsive disorder [cases 14, 15]. In the patients with schizophrenia [3, 6] the accent change co-occurred simultaneously with a psychosis. The patients who did not suffer psychiatric symptoms, related the onset of their FAS to a motor vehicle accident [cases 1, 11], a “near-accident” [case 5], possibility of MS [case 2], a whiplash trauma 9 years prior to consultation for FAS *or* after consultation of an otolaryngologist for a change of voice quality after a minor head trauma [case 4], admission to hospital for the sudden onset of sensory and gait symptoms [cases 9, 10, 12]. In 47% of the FAS cases considered psychogenic, the onset of the accent was delayed in comparison to the occurrence of the adverse life event that was held responsible for the FAS by the patients themselves [cases 2, 4, 5, 9–12]. In 5 of these cases, the patients were diagnosed with a conversion disorder [cases 5, 9–12].

In 27% of the cases (*n* = 4/15) [cases 3, 6, 7, 13], the accent resolved simultaneously with the associated psychiatric disorder. In two cases (13%) [cases 4, 10] FAS resolved spontaneously. In all other patients [1, 2, 5, 8, 9, 11, 12, 14, 15], FAS remained present throughout follow-up. In case 5, scores on the Minnesota Multiphasic Personality Inventory (MMPI; Butcher et al., 1989) and Dissociation Questionnaire-Revised (DISQ-R; Vanderlinden et al., 2009) were *near* the accepted mean, but the accent persisted.

Only three patients received speech-language therapy to reduce FAS [cases 4, 10, 12]. Van Borsel et al. (2005) applied auditory masking and delayed auditory feedback (see also comments of Moreno-Torres et al., 2013). However, these interventions did not resolve FAS. Case 10 received a symptomatic intervention for psychogenic voice and speech disorders (Duffy, 2005). However, progress did not transfer to conversational speech and the accent suddenly resolved after having quit outpatient therapy for several weeks. Case 12 agreed to behavioral speech therapy as well (targeting the production of individual speech segments), but she quit after one session for reasons that were not disclosed.

For patients whose accent change *resolved* during follow-up [cases 3, 4, 6, 7, 10, 13], the period between accent onset and remission was about 63 days on average, i.e., 9 weeks (range: 6 days–6 months, SD: 71 days). The patient described by Reeves and Norton (2001) [case 3], was re-admitted to hospital three times and this was taken into account for the calculation of the duration. In 60% of the cases [cases 1, 2, 5, 8, 9, 11, 12, 14, 15] the accent did *not* resolve. In these patients, investigation of the period between accent onset and last follow-up revealed that the accent persisted for 45 months on average^2^ (range: 15 months–8 years; SD: 28 months and 2 days).

### Psychodiagnostic and neuropsychological testing

Formal psychodiagnostic testing was carried out in three patients (see Table 4). In case 5, the results obtained on the MMPI-2 in 1995 showed a conversion V-pattern. The conversion V-form designates a markedly low score on the depression scale (scale D): the conversion suppresses depression, which explains lower scores on scale D. On the other hand, it is associated with increased physical sensations, thereby increasing scores on the hypochondriasis scale and hysteria scale (Leavitt, 1985). The second patient's profile elicited an elevated degree of defensiveness (K: 70) and hysteria (Hys: 61). The restructured clinical scales revealed marginally elevated scores for depression (RC2: 66) and somatic complaints (RC1: 57). The elevated scores on the hysteria scale in conjunction with the somatic complaints (although only marginally elevated) are additional arguments to suspect conversion disorder, though the typical V-pattern was not found. Although exact scores were not provided, a conversion-V profile was also found on the MMPI-2 for case 12 (code type 1-3/3-1 is generally associated with conversion disorder). Scores on the neuroticism scale of the NEO-PI-R were low, which indicates stable personality and emotions, calmness, but also a decreased reactiveness to everyday situations (Nelson, 2014). The patient scored in the average range for the extraversion, agreeableness and conscientiousness scales. No mention was made of scores for openness to experience. The SCL-90-R is a “90-item self-report symptom inventory” (Derogatis and Savitz, 1999) in which the patient rates the severity of a series of psychiatric symptoms. These are grouped around nine dimensions: somatization, obsessive-compulsiveness, interpersonal sensitivity, depression, anxiety, hostility, phobic anxiety, paranoid ideation, and psychoticism (Domino and Domino, 2006). Only one clinical score was mentioned, i.e., for the somatization scale (*T* = 65). This agrees well with the profile elicited on the MMPI-2. The STAI is a self-report scale for anxiety consisting of two 20-item scales. The patient indicates (1) how he/she feels now (*state*) and (2) how he/she feels generally (*trait*) (Lam et al., 2005). Scores on the STAI were subclinical. Finally, the BDI-2 is a self-report inventory, which consists of a series of statements concerning complaints. The patient notes how he/she feels about the statements taking into account his/her psychological status over the last week. Scores on the BDI-2 were equally sub-clinical.

**Table 4:** **Overview of the patients subjected to psychodiagnostic tests**.

**Psychodiagnostics**	
**Test**	**Case number(s)**
MMPI-2 (Butcher et al., 1989)	5, 11, 12
DISQ-R (Vanderlinden et al., 2009)	5
BDI-2 (Beck et al., 1996)	12
NEO-PI-R (Costa and McCrae, 1985)	12
SCL 90-R (Derogatis, 1983)	12
STAI (Spielberger et al., 1970)	12

MMPI-2, Minnesota Multiphasic Personality Inventory-II; DISQ-R, Dissociation Questionnaire Revised; BDI-2, Beck Depression Inventory-2; NEO-PI-R, Neuroticism Extroversion Openness Personality Inventory, Revised; SCL-R, Symptoms Checklist-90-items, Revised; STAI, State Trait Anxiety Inventory.

Only in a small number of case studies formal neuropsychological investigations were carried out. General cognition, memory, attention, executive functioning, and language was assessed in 4 cases [cases 5, 8, 11, 12]^3^ (see Table 5).

**Table 5:** **Overview of the patients subjected to neuropsychological tests**.

**Neuropsychology**	
**Test**	**Case number(s)**
**GENERAL COGNITIVE SCREENING TESTS**	
MMSE (Folstein et al., 1975),	5
CLQT (Helm-Estabrooks, 2001)	10
WRAT (Wilkerson, 1993)	11^*^, 12
**INTELLIGENCE**	
WAIS (Wechsler, 1981, 1997a)	5, 9, 11, 12
**MEMORY**	
WMS (Wechsler, 1991, 1997b)	5, 12
Brown Peterson Task (Brown, 1958)	8
CVLT (+learning) (Delis et al., 2000)	12
RAVLT (+learning) (Rey, 1941)	11
BVMT-R (Benedict, 1997)	12
**ATTENTION, SET-SHIFTING**	
Stroop task (Stroop, 1935)	5, 8, 11, 12
Ruff figural fluency (Ruff, 1988)	12
TMT (Reitan, 1958, 1992)	5, 8, 11, 12
**VISUO-SPATIAL ABILITIES**	
Rey complex figure (Rey, 1941)	5, 11
Judgment of line orientation (Benton et al., 1983)	5
**MOTOR FUNCTIONING**	
Finger tapping test (Arnold et al., 2005)	11, 12
Grooved pegboard (Kløve, 1963; Lafayette Instrument, 2002)	12
**SYMPTOM VALIDITY TESTS**	
Green word memory test (Green, 2005)	12
**DEMENTIA SCALES**	
HDS (Cole et al., 1983)	5
ADAS (Rosen et al., 1984)	5
**LANGUAGE**	
BNT (Kaplan et al., 2001)	3, 6, 7, 10–12
PPTT (Howard and Patterson, 1992)	8
Token Test (De Renzi and Vignolo, 1962)	4, 8, 12
BDAE (Goodglass et al., 2001).	2^*^, 3, 5, 10
AAT (Graetz et al., 1992: Dutch version)	4^*^, 5
MAE (Benton et al., 2001)	10^*^, 11^*^, 12^*^
SAN-TEST (Deelman et al., 1981)	4^*^
DO-80 (Deloche and Hannequin, 1997)	8
Picture naming via an experimental test	2
PENO (Joanette et al., 1990).	8^*^
Phonemic fluency (FAS) (Norms: Tombaugh et al., 1999, case 11; Benton et al., 2001: case 12; case 5: unpublished norms)	5, 11
semantic fluency (animals, transport, vegetables, clothes: unpublished norms)	5
Word/sentence reading via an experimental test	2
Word sentence spelling via an experimental test	2

MMSE, Mini Mental State Examination; WAIS, Wechsler Adult Intelligence Scale; WMS, Wechsler Memory Scale; TMT, Trail Making Test; WRAT, Wide Range Achievement Test; CVLT, California Verbal Learning Test; RAVLT, Rey Auditory Verbal Learning Test; CLQT, Cognitive Linguistic Quick Test; BVMT-R, Brief Visuospatial Memory Test-Revised; HDS, Hierarchic Dementia Scale (HDS); ADAS, Alzheimer's Disease Assessment Scale; BNT, Boston Naming Test (BNT); PPTT, Pyramid and Palm Tree Test; MAE, Multilingual Aphasia Examination; BDAE, Boston Diagnostic Aphasia Examination; AAT, Akense Afasie Test (Dutch version); SAN-test, Stichting Afasie Nederland; DO-80, Test de Dénomination Orale d'Images; PENO, Protocole d'Evaluation Neuropsychologique Optimal.

2* : possibly only two subtasks of the BDAE were administered: the non-verbal and the verbal agility test.

4* : only written language via AAT; sentence comprehension and word retrieval (animals) SAN-Test.

8* : letter and category fluency.

10* : auditory word and sentence comprehension, sentence repetition, and oral and written spelling MAE.

11* : word reading and spelling tests of the WRAT; sentence repetition task, as well as the aural and reading comprehension task MAE.

12* : repetition skills, auditory comprehension, token task, and reading comprehension MAE.

In case 9, only intelligence was investigated. In cases 3, 4, 6, 7, and 10 only language testing was performed. Neuropsychological examination consisted of a variety of tests (Table 5).

Cognitive performance was “within normal limits” (p. 715, Gurd et al., 2001) for case 2 and average to above average on all tasks in case 5. In case 8, memory and attention were normal, but the patient gave evidence of difficulties with short-term memory (Brown Peterson Task: mean of interference scores: 42%; norm: 97.22%, SD: 4.46), as well as with attention control and executive functions (Stroop test: Stroop effect: 249″, norm: 142.4″, range: 88–204″; TMT-A: 61″, norm: 41.3″, SD: 15″ and TMT-B: 253″, norm: 111.4″, SD: 72.2″). In case 9, results on the WAIS-R were within the normal range (VIQ = 96, PIQ = 107, and FSIQ = 101). Case 11 presented poor executive functions (Stroop test, Interference < 1 pc., and TMT-B: 83″, mean: 56.0, SD: 21.2), problems with attention and poor processing speed (TMT-A: 43″, mean: 23.8, SD: 6.9, Stroop test A: 101″, < 1 pc.). Case 12 demonstrated impaired intelligence, memory, attention, executive functions and fine-motor skills: WAIS-III (FSIQ = 65, VIQ = 76, PIQ = 60); Trail Making Test (146″), Grooved Pegboard (dominant hand: 149″, mean = 85″, range: 48″–121″, non-dominant hand: 130″, mean = 101″; range: 47–152″), and Green Word Memory Test (Green Word Memory Test: immediate = 87.5, delayed = 77.5, consistency = 70.0).

Most patients in whom language was assessed, obtained average to above average results [cases 3–7, 10]. Case 2, however, had impaired oral agility as demonstrated by the BDAE (non-verbal agility: 4/12 and verbal agility: 7/12). Case 8 presented with (severely) depressed scores on phonemic and semantic category fluency (letter fluency: 5, mean: 45.46, SD: 16.4; category fluency: 14, mean: 47.85, SD: 9.8). Case 11 obtained depressed scores on most tasks evaluating speech and language (WRAT; reading: 43, pc. 6; spelling: 43, pc. 37); MAE sentence repetition (A: 2, < pc. 1 and B: 3, < pc. 1), verbal fluency (FAS): 19, pc. 2. Case 12, also demonstrated low average to impaired scores on most of the administered tasks: the BNT score was considered low average (41/60). On the MAE the following scores were obtained: repetition: 5 (impaired); auditory comprehension: 15 (borderline impaired), token test (as part of MAE): 40 (low average), and reading comprehension: 16 (borderline).

### Comorbid speech and language disorders

Five cases presented additional speech and/or language deficits [cases 4, 5, 8, 11, 12], apart from FAS. Case 4 (Van Borsel et al., 2005) and case 12 (Jones et al., 2011) went through a period of pre-FAS mutism. In case 4 mutism was only documented by self-report. Van Borsel et al. (2005) noted that the patient's language was characterized by grammatical anomalies. This was also the case for the patient of Poulin et al. (2007) [case 8].

Case 5 implemented French syntax in native Dutch speech. Non-fluent expressive output was characterized by mistakes typically made by French learners of Dutch. Oral output of case 11 was initially considered as dysarthria, later as “apraxia of speech” (p. 1010). As mentioned, the patient obtained lower scores for verbal fluency (F,A,S), but also for sentence repetition (MAE A&B: pc. < 1) and the reading and spelling tasks of the WRAT (reading: 43, pc. 6; spelling: 43, pc. 37). It could have been expected that these symptoms are related to neurological damage. Indeed, apraxia of speech is caused by structural damage to the anterior insula of the language dominant hemisphere (Dronkers, 1996). Nevertheless, contrary to expectations, repeat structural imaging of the brain (CT and MRI) did not disclose any damage. In addition, FAS was accompanied by “telegraphic speech” (irregularly deleting prepositions, for instance). In this particular case, the comorbid symptoms and the language deficits were regarded as “not credible” because the extent of the deficit did not correspond to neuroimaging findings. The patient was diagnosed with FAS of a non-organic nature because of inconsistencies in the language symptoms.

## Discussion

### Demographic data

Analysis of the available literature suggests that psychogenic FAS is quite rare (*n* = 15/105) (14%). During the past decade FAS has increasingly attracted the attention of the scientific community as 93% of the psychogenic FAS cases (n = 14) were published in a time span of only 12 years (2001–2013). The finding that there are more women with psychogenic FAS than men (67% are women, 33% are men), might be partly explained by the increased predisposition of women to several of the associated psychopathologies. Most mental disorders are also more prevalent among women than men (see also: World Health Organization, 2014). For schizophrenia, prevalence figures are esteemed to be equal, irrespective of gender, though symptoms occur earlier in men (Angermeyer and Kühnz, 1988; Saha et al., 2005; National Institute of Mental Health, 2015). On the other hand, the analysis of the neurogenic population revealed a similar demographic distribution: 68.6% of the authentic (neurogenic) FAS cases were women (*n* = 59/86). Interestingly, Baker (2003) points out that it should also be taken into account that women are twice as likely to seek medical attention than men. It thus seems that the explanation for this demographic distribution remains speculative.

### Associated psychopathologies

Several different psychopathologies have been associated with FAS. In patients with schizophrenia, all FAS episodes *co-occurred* with a discontinuation of anti-psychotic drugs, which caused exacerbations [cases 3, 6]. In the bipolar patients FAS also co-occurred with positive symptoms [cases 7, 8]. Reeves et al. (2007) put forward the hypothesis of a direct link between the manic/psychotic exacerbations and FAS in their patients via a Positive And Negative Syndrome Scale (PANSS; Kay et al., 1987). They also suggested that FAS could have been related to a temporary disruption of the inhibition of the bilateral superior temporal gyri (STG) during exacerbations. The STG is inhibited in healthy controls when the left dorsolateral PFC is activated for word generation. It is hypothesized that FAS may have been caused by the intermittent suppressed neural circuitry.

Moreno-Torres et al. (2013) observed that the dopaminergic system may be disrupted in FAS patients. The intake of dopamine antagonists (olanzapine, risperidone) in case 3 and 6 could have restored the neurotransmitter balance and diminish the FAS. Particularly in schizophrenic patients, the so-called “dopaminergic hypothesis” (Meltzer and Stahl, 1976; McCutcheon and Stone, 2015) agrees well with this theory. This hypothesis claims that positive symptoms in schizophrenia can be reduced by the intake of dopamine antagonists or dopamine D2-receptor blockers. It has also been shown that modulation of the dopaminergic system influences the functionality of the (pre)fronto-striato-pallidal-thalamic network, which is hypothesized by Reeves and Norton (2001) to be implicated in the accent change, and has been related to the occurrence of psychosis (Honey et al., 2003).

The symptoms of case 13 might be explained along the same lines, as excess dopamine transmission has been suspected to incite manic symptoms (Swerdlow and Koob, 1987; Cookson, 2013). Nevertheless, the pathophysiology of both psychiatric disorders is characterized by subtle differences. In schizophrenia, abnormal activity occurs in the striatum and the prefrontal cortex, whereas in mania the activity may be located more toward the dorsal nigrostriatal pathways (Cookson, 2013). Nevertheless, Cookson (2013) reported that antipsychotic drugs such as risperidone, and olanzapine (dopamine antagonists, and more specifically the ones administered to the schizophrenic FAS cases: case 3 and 6) work well on manic symptoms, such as pressured speech. The speech of case 13 was marked by excessive pressure, increased speed, loudness and forcefulness. The patient's FAS resolved simultaneously with resolution of mania after pharmacological treatment.

In case 8, a psychiatrist related the accent change and sudden Spanish and German sounding words to a psychological problem at a subconscious level. Poulin et al. (2007) performed a ^18^F-FDG-PET scan which demonstrated metabolic changes in the area of the left insular and anterior temporal cortex and a diffuse hypoperfusion affecting the frontal, parietal, and temporal lobes bilaterally. MRI of the brain showed a slight asymmetrical atrophy. All imaging was performed in euthymic state. The possibility that both the language and psychological disorder were consistent with the neuroradiological findings was considered. However, the alterations at a linguistic level remain odd, even in the light of the attested neuroradiological findings. For instance, the output of the patient—contrary to what is expected in cases of agrammatism—was fluent, and despite a hypoperfusion affecting the insula, articulation was perceived as normal in every respect. There was no sign of apraxia of speech-, dysarthria-, or aphasic-like symptoms. All of the investigated linguistic functions were normal, except for a deficit in letter and category fluency.

Case 14 and 15 suffered from refractory OCD and were treated by means of deep brain stimulation (DBS). They both developed hypomanic behavior and started experiencing accent changes afterwards. The hypothesis of FAS due to an undetected lesion induced by the electrode implantation was excluded, as the accent only developed *after* the actual stimulation by the electrode and post-operative CT confirmed the absence of any additional structural brain damage. Furthermore, Polak et al. (2013) argue that lesions caused by DBS are smaller than those generally associated with FAS, including the peri-sylvian area, (pre-)motor area, and insula of the language dominant hemisphere. However, dysfunction of the previously mentioned cortico-striato-pallidal-thalamic loop has frequently been suspected to be the pathogenic mechanism behind OCD, and the function of this circuit is altered when the nucleus accumbens is targeted for DBS.

“Hysteria,” or “hysteric reaction,” the term Whitaker (1982) used as an exclusion criterion for FAS, is an outdated term for “conversion disorder” [cases 5, 9–12]. Conversion disorder has been subsumed under the concept of “*hysterical* neuroses” in the DSM-II [American Psychiatric Association (APA), 1968]. According to Aronson and Bless (2011) a conversion reaction can affect any system requiring sensory or voluntary motor control and hence, also voice and speech. DSM-IV-TR [American Psychiatric Association (APA), 2000] criteria allow for such an interpretation as well, although the concept has frequently been the object of debate and is regarded insufficiently clearly defined to allow for a conclusive diagnosis (e.g., Delis and Wetter, 2007; Stone et al., 2011). In all psychogenic FAS patients with conversion disorder or those patients for whom the hypothesis of a conversion disorder was raised, the shift in accent was never the “first” conversion symptom to occur: all case studies report more general physical discomforts that *preceded* the FAS. Especially gait and balance disturbance [cases 5, 9, 10–12] occurred but also a range of sensory problems including tinnitus [case 9], left-sided weakness affecting face and arm [case 10], blurred vision [case 10], altered hearing [case 10], abnormal sensations in arms and legs [case 10], facial numbness [case 11], weakness in the right arm [case 11], deafness to the left ear [case 11], give-way weakness [case 12], and a right-side sensory loss [case 12].

In cases 2 and 4 an associated psychological disorder was not obvious, rather there was a range of clinical observations and findings from radiological and neurophysiological investigations, which suggested a potential psychogenic origin of FAS. Gurd et al.'s patient (2001) [2] was qualified as “psychogenic,” even though CSF analyses revealed oligoclonal bands, a bio-marker of Multiple Sclerosis (MS) and EEG revealed transient spikes over the left temporal lobe. T2 hyper-intensities were found on MRI (judged clinically insignificant). It is therefore questionable whether patients suffering from MS (Gurd et al., 2001; Villaverde-González et al., 2003; Bakker et al., 2004; Chanson et al., 2009) really develop FAS as a consequence of their neurological disorder or due to accompanying psychological distress. Grazioli et al. (2008) note that over 50% of the MS patients suffer from depression. Case 2 obtained borderline results on the Hospital Anxiety and Depression Scale (Zigmond and Snaith, 1983). The case of Bakker et al. (2004) was noted to have very “labile emotions” (p. 271). The case of Villaverde-González et al. (2003) had a history of depression as well as an elevated irritability (p.1035). For the other patients, psychological well-being was not indicated.

Van Borsel et al.'s (2005) patient [case 4] had no demonstrable lesions on CT, and displayed no symptoms apart from a change of accent and some articulatory and grammatical difficulties. She had sustained a head trauma and whiplash 9 years earlier and had suffered from chronic headaches ever since. Her accent change had occurred after a visit to the otolaryngologist, approximately 1 month after she had suffered another minor head trauma. Van Borsel et al. (2005) diagnosed the speech disorder as non-organic FAS because of a psychiatric history (depression and suicidal ideation) which was related to marital problems, a completely normal neurolinguistic assessment apart from mild grammatical anomalies, articulatory difficulties, and an accent change, the absence of a organic deficit, and a spontaneous resolution of the accent 5 months after the initial visit.

Case 11 suffered a minor head trauma as well but developed FAS only 3 years later, associated with intermittent, atypical expressive language deficits, and apraxic as well as dysarthric symptoms. Initially, she also claimed that she was deaf to her left ear, but a hearing loss was formally ruled out. The patient displayed an “inconsistent” agrammatism, characterized by deletions of function words. She would use and subsequently erase the same words in a series of successive utterances. She also made other inconceivable mistakes, such as splitting numbers into digits. Given the high degree of automaticity of such numerical output, these errors are highly unlikely to occur in the absence of other language deficits. Since she passed most of the symptom validity tests, she was considered not to be feigning or malingering and was ultimately diagnosed with conversion disorder.

### Segmental and suprasegmental characteristics

Patients with FAS of an assumed psychogenic etiology present with a variety of segmental and suprasegmental errors. At the segmental level, the image more or less corresponds to what is generally found in neurogenic patients, including a dissociation between vowels and consonants (e.g., Katz et al., 2008). At the suprasegmental level, slow speech rate is often seen [cases 5, 8, 10–12]. Slow speech rate can be linked to slow processing speed, which may occur as a consequence of psychological and psychiatric impairment (e.g., depression, post-traumatic stress disorder, bipolar disorder, and schizophrenia). Analysis of (psychogenic) FAS-related segmental and suprasegmental errors has been predominantly impressionistic, except for a few cases in which (acoustic) measurements (e.g., fundamental frequency, speech intensity, speech, and articulation rate) were also included [cases 5, 8, 10, 12, 13]. Deviant intonation [cases 3, 6–13] is a function of pitch variation. Intonation was off in most patients with a reduced speech rate [cases 8, 10–12], but also in patients who spoke at a normal or even fast pace [case 13]. In four cases [cases 3, 6, 7, 13], deviant intonation may be associated with a psychopathology. In schizophrenia [cases 3, 6], difficulties with receptive affective prosody have been described (Rossell et al., 2013). However, Hoekert et al. (2007) state that dysfunctional *expressive* affective prosody also qualifies the speech profile. The manic patient of Lewis et al. (2012) demonstrated fast speech [FAS: 229 wpm; base line speech (BL): 173.9 wpm; average speech rate: 190 wpm based on (Yorkston et al., 1996)] and a pitch level that was considerably higher during FAS than during the baseline condition (conversational speech; FAS: 265.63 Hz, BL: 160.56 Hz; average F0 for a woman: 160–225 Hz based on Baken, 1987; Titze, 1994) (see also: Hanwella and de Silva, 2011). A higher speech rate was negatively correlated with the size of the vowel space, i.e., a higher speech rate leads to a more compressed vowel space in non-brain damaged subjects, which was exactly what Lewis et al. (2012) found in their patient. This compression could explain the reduced intelligibility of speech in comparison to the BL conversation sample (FAS: 73% vs. BL: 100% intelligible): contrasts between vowels diminish and vowel duration is shortened (Chen et al., 1983; Turner et al., 1995; Weinrich and Simpson, 2014).

### Accent change

The overview of the different accents of the analyzed cases shows that there does not seem to be any consistency. However, some interesting observations can be made. Firstly, it is striking that in 7 out of 15 cases (47%) the accent changed from the standard language variant to a regional one, or the other way round. In 9 cases (60%) the mother tongue was some variant of English: either British English [cases 1, 2] or American English [cases 3, 6, 7, 10–13]. FAS is frequently documented in Anglo-saxon media^4^, as such the syndrome is more commonly known among lay people. For some cases more than *just* the accent gave the listeners the impression of a very specific foreign accent: language mixing (e.g., case 6) and code switching [case 3, 14, 15] were also observed. Code switching can be defined as switching between language varieties or registers within a single conversation. For case 3, this involved the use of words such as “*blokes*” instead of the usual American variant “*friend.*” Case 14 occasionally^5^ used a dialectal variant of Dutch while case 15 employed a vocabulary typifying a more formal register, e.g., the patient used words such as “*public toilet*” instead of the more informal: “*loo.*” Polak et al.'s (2013) patients' alterations could be related to DBS, as such linguistic modifications can occur after stimulation. Verhoeven et al.'s (2005) 51-year-old female patient (case 5) occasionally used French words, made literal translations from French to Dutch, and adapted syntactic structures resembling Dutch of second language learners. It has to be mentioned that this patient had been a teacher of Dutch in a French company based in Holland and this may have rendered her very conscious of mistakes generally made by French learners of Dutch. These symptoms constitute another point of difference between the neurogenic and psychogenic patient population, as the insertion of foreign words or regional expressions was previously only noted in a case of Ryalls and Whiteside (2006: insertion of British equivalents of American expressions) and a case of Laures-Gore et al. (2006, case 2: insertion of Spanish words in English speech). Both case reports, however, represent instances of mixed FAS (see also Verhoeven and Mariën, 2010). “Pure” neurogenic FAS patients who demonstrated such lexical borrowings have not been identified.

### Psychodiagnostic and neuropsychological testing

Only three patients were tested with formal psychodiagnostic test batteries. Only in two patients [case 5, 12] the pattern was significant for a conversion disorder. In case 11, somatization and hysteria were (slightly) elevated and a diagnosis of conversion disorder was agreed upon based on the inexplicable symptom course and the presence of symptoms which could not be explained on the basis of neurological impairment (apart from the FAS, sensory and motor problems equally occurred: see also Section Associated Psychopathologies). For case 9, who underwent a psychodiagnostic interview, family conflict was regarded to have had such a profound effect on the patient's mental state, that the symptoms could be related to psychological problems and a childhood trauma.

Only for case 11, additional symptom validity tests were administered. Incorporation of these tests in psychodiagnostic testing is always recommended, not only when secondary gains are at stake [case 11], but also when the impact of traumatic experiences or psychological discomforts are (possibly) downplayed (Cima et al., 2003; Bush et al., 2005). In these cases, it is important to interpret neurocognitive test results with caution, as these too can be consciously manipulated (see also: “cogniform condition/disorder”: a recently developed concept within the somatoform disorders; described by Delis and Wetter, 2007).

With respect to neuropsychological testing, results were diverse for scores on tasks evaluating memory, intelligence, executive functions and attention. Three out of the five patients diagnosed with conversion disorder had poor memory and/or attention and executive functions [cases 8, 11, 12] and in one instance, deficits in fine motor skills were also observed [case 12]. Deficits in learning and memory, but also in executive function, attention, processing skills and word finding have been associated with somatoform disorders (Niemi et al., 2002; Trivedi, 2006; Demir et al., 2013). Especially, attention and executive functions are often impaired in this patient group. One of the hypotheses that have been raised to explain cognitive impairment in this group is that these deficits relate to frontal brain dysfunction. However, Wall et al. (2013) point out that the studies claiming an association between cognitive deficits and conversion disorder did not include symptom validity tests in their test protocol for patient selection and therefore no generalizations can be made. Still, the authors argue that the incidence of neurologically inexplicable cognitive deficits in patients with conversion disorder is quite high. It remains unclear whether there is a fixed set of neurocognitive deficits specific to this population, or, as others argue, whether the deficits are related to the associated psychiatric distress (Lamberty, 2008).

### Remission of the FAS

In the neurogenic population a late onset of FAS has only been noted when the FAS was “masked” by other speech or language disorders (mutism, Broca aphasia, apraxia of speech, or dysarthria). Apart from a pre-FAS muteness [cases 4, 12] and apraxic/dysarthric-like symptoms in one case [case 11], FAS was never “masked” by preceding speech/language deficits in current group. Hence, a delayed onset might be indicative of a psychogenic origin. For 27% of the investigated patients (*n* = 4/15), FAS resolved simultaneously with the remission of the related psychopathology [cases 3, 6, 7, 13]. In those cases, FAS developed after psychosis or after a (hypo)manic attack and was associated with a sudden withdrawal of neuroleptic drugs, or an unbalanced drug intake. In two cases (13%), FAS resolved spontaneously [cases 4, 10]. Only three patients received speech-language therapy in order to reduce the FAS [cases 4, 10, 12], and case 11 received speech-language therapy before the accent appeared. Case 10 received the symptomatic speech therapy as proposed by Duffy (2005). According to the authors, the patient occasionally managed to accurately realize the target items, though she herself did not embrace her progress. Delayed auditory feedback and auditory masking did not improve the speech deficits in the patient reported by Van Borsel et al. (2005), although this approach has been advocated by other researchers as well (González-Álvarez et al., 2003; Moreno-Torres et al., 2013). Butcher et al. (2007) point out that there is a lack of evidence-based treatment strategies for psychogenic speech and language disorders, and that this is directly related to the uncertainty and lack of confidence on the part of the speech therapist to diagnose a disorder of psychogenic origin. To the best of our knowledge, no large-scale study has ever been carried out to evaluate the effectiveness of a treatment for psychogenic *speech* disorders.

### Comorbid speech and language deficits

Table 1 shows that two patients [cases 4, 12] were mute before the onset of FAS. Psychogenic mutism is well-recognized [Salfield, 1950; DSM-V: American Psychiatric Association (APA), 2013]. For case 4, the mutism can be related to the impact of psychological issues (depression, suicidal ideation) as well as to severe anxiety problems (permanent fear that the patient's son might develop Huntington disease). Case 12 was diagnosed with a conversion disorder. Mutism has previously been diagnosed in patients with conversion disorder and, in those specific cases, it is also referred to as “conversion mutism” (Rothbaum and Foa, 1991; Aggarwal et al., 2010).

In three cases, language was also characterized by agrammatic output [4, 8, 11]. McKenna and Oh (2005) note that Karl Kleist as early as 1914, used both the terms agrammatism (non-fluent, as in Broca-like speech; mostly seen in catatonic patients) and paragrammatism (fluent, more as in Wernicke-like speech; mostly seen in paranoid patients) in a psychiatric context. In 1976, Norman Geschwind described the case of a patient with a “hysterical pseudo-agrammatism” (Geschwind, 1976). The patient had been locked up in prison for passing bad checks, after which he suddenly developed a strange speech disorder and was admitted to a mental institution. What struck Geschwind was that the patient produced agrammatic speech at a normal rate in combination with stuttering behavior, a combination of symptoms, which according to Geschwind was “unique” (p. 81) and very unlike what is seen in agrammatic aphasic patients. In 1983, Levy and Jankovic published an experiment, in which they induced a (placebo) conversion reaction in a female patient in her mid-twenties. The researchers set up a double-dissociation experiment: first, the patient received a saline injection, but she was told it contained phenytoin. Later, she received the phenytoin injection, but this time she was told it contained “a neutral substance.” The patient's neurological symptoms worsened after each explicitly mentioned “raise” in phenytoin, as did her scores on the various neurolinguistic exams (among others: the BDAE; Goodglass and Kaplan, 1972). Her speech became slower, (moderately) slurred and hypophonic. She made several literal paraphasias, used a telegrammatic style in repetitions and spontaneous speech, and employed overgeneralizations in picture naming. After the medicine was told to “have worn off” completely, neurolinguistic testing demonstrated only one (!) naming error. De Letter et al. (2012) reported three cases with (non-fluent) agrammatism, overgeneralizations, and paraphasias which could not be attributed to an underlying organic cerebral pathology. All three patients presented with psychiatric conditions: case 1 suffered from bipolar disorder, case 2 had a “manipulative personality” (p. 877), and case 3 had quite an extensive psychiatric history marked by mood swings, depression, and aggressiveness. All patients produced non-fluent speech, characterized by excessively long pauses. Furthermore, the patients demonstrated hypophonia, persevered in their errors, and spoke with a reduced speech rate. As was the case for the patient of Levy and Jankovic (1983) the patients never produced frustrated reactions and never attempted self-correction. For De Letter et al. (2012) the fluctuating language problems and neurological symptoms were the primary reasons for considering the speech/language problems of their patients as psychogenic, although they demonstrated organic anomalies. They argue that “the presence of a language disorder in patients with organic cerebral disease cannot demonstrate causation (e.g., Whitlock, 1967)” (p. 876).

Van Borsel et al. (2005) explicitly argues that “grammatical anomalies […] did not conform to the pattern of agrammatism typical of Broca's aphasia or paragrammatism as seen in Wernicke's aphasia” (p. 424). In case 8, the agrammatism was equally noted in a context of otherwise well-articulated, fluent speech. However, apart from verbal fluency deficits (category and letter fluency) in case 8, there were no other notable deficits that characterized the neurolinguistic profile of most of these agrammatic patients. For case 11, it was mentioned that the patient had an agrammatism that was typologically different from Broca-aphasia (Kean, 1977, 1985): e.g., the patient was fluent and speech was not consistently agrammatic as she was able to rephrase sentences, and use initially omitted prepositions or verbs.

The case described by Cottingham and Boone (2010) [case 11] also presented with dysarthria-like symptoms and a suspected apraxia of speech, for which no structural lesions were seen on CT or MRI. Hence, the speech and language symptoms of their patient were considered as “non-credible.” There are other reports of patients demonstrating similar incredible language symptoms. Recently, a report of De Witte and Mariën (2015) observed inexplicable post-operative language symptoms and considered them as psychogenic in a 28-year-old male patient, who had undergone awake surgery for the removal of a tumor in the left anterior inferior temporal gyrus. Post-operatively, the patient was able to repeat, read, write, name high and middle frequency words but auditory comprehension and naming of low frequency words were severely impaired and he displayed inconsistent comprehension deficits. It was noted that results on the CES-D (Center for Epidemiological Studies Depression; Eaton et al., 2004) and STAI (Spielberger et al., 1983) were higher than the cut-off, indicating a higher risk for depression or anxiety disorder. De Witte and Mariën (2015) hypothesize that the symptoms of their patient were non-organic because of the patient's sensitivity to stress and depression, the atypical (course of the) symptoms, and the fact that, despite the comprehension deficits, the patient had very good insight in the disorder as his aunt suffered from vascular aphasia. If the symptoms themselves, or the course of the symptoms, cannot be explained by attested neurological deficits, the possibility of a psychogenic etiology should at least be considered (see also: Baumgartner, 1999).

The case reported by Verhoeven et al. (2005) [case 5], presented with a form of “pseudo-paragrammatism.” This patient's speech was characterized by mistakes typically made by French learners of Dutch. The patient did not speak in a telegram style speech, nor did she omit function words. She did, however, change the syntax in such a way that it no longer corresponded to what could be expected in her native language. She used French grammar in Dutch discourse, but not when speaking English. Paragrammatic speech is generally fluent, and marked by complex sentences which contain function words, verbs (also finite ones), nouns, in short: all elements required for the construction of a well-formed sentence are present, but the speakers do not apply the grammatical rules as expected.

## Shortcomings and limitations

The results of this review should be interpreted with caution. The scarcity of comparable measures characterizing the case reports compelled us to limit the quantitative analysis of FAS. With a view to future diagnostics, it is hoped that linguistic manifestations, medical findings, medical history, and psychiatric symptoms are documented in great detail, in order to enable a reliable FAS diagnosis and suitable therapeutic interventions.

## Conclusion

This paper explored psychogenic FAS as a subtype of FAS. The following conclusions can be drawn: firstly, psychogenic FAS is related to the presence of a psychiatric or psychological disturbance in the absence of demonstrable neurological damage or an organic condition that might explain the accent. Secondly, psychogenic FAS occurs more in women than men, in an age range which is likely to be prone to depression and mental problems (25–49 years). Thirdly, psychogenic FAS is characterized by both suprasegmental and segmental changes. A deviant intonation (variable pitch) and a slow speech and articulation rate are the most typical prosodic features. At a segmental level, vowels are more affected than consonants. Future research should report on segmental and suprasegmental changes in as much detail as possible, in order to aid diagnosis based on semiological distinctions between neurogenic and psychogenic FAS. Fourthly, the remission of FAS seems to be related to resolution of comorbid positive psychiatric symptoms. Fifthly, psychodiagnostic testing—including symptom validity tests—is highly recommended with a view to suspected psychogenic FAS; not only in view of adequate therapy, but also for the interpretation of cognitive deficits, which may be aggravated as well. Sixthly, patients with psychogenic FAS often demonstrate linguistic features in speech and language that are not consistent with neurogenic speech/language disorders, e.g., in psychogenic cases, FAS can co-occur with a form of isolated “pseudo-” agrammatism in unaffected fluent speech (different from agrammatism seen in non-fluent aphasic patients) and paragrammatism. Pre-FAS mutism has also been attested. Furthermore, language often shows code switching and language mixing which rarely occurs in polyglot aphasic patients.

Future research should work toward validation of a set of criteria for psychogenic FAS via an extensive comparison with the neurogenic cognate. Moreover, in view of an efficient therapeutic guidance and clinical diagnosis, future research should focus on the treatment of non-organic *speech and language* disorders in large populations. We believe that a combination therapy focusing on the cognitive-behavioral problems on the one hand, and the speech and language deficits on the other, may be beneficial in this population. The intricate symptomatology often gives proof of overlapping cognitive, psychological and speech problems, and the FAS is interpreted as an (indirect or direct) emanation of the underlying psychological disturbances.

## Author contributions

Conception and design: SK, PM, JV, EDW; acquisition of data: SK, PM, JV, EDW; analysis and interpretation of data: SK, PM; drafting of the manuscript: SK and PM; critical manuscript revision: all authors; and final manuscript approval: SK and PM on behalf of all authors.

### Conflict of interest statement

The authors declare that the research was conducted in the absence of any commercial or financial relationships that could be construed as a potential conflict of interest.

## Footnotes

^1^The case reported by Reeves and Norton (2001) was reported again in Reeves et al. (2007; case 3) and the case reported by Poulin et al. (2007) is identical to the case reported by Roy et al. (2012, case 1). However, all the available information was used for further analyses.

^2^The exact duration is unknown. The calculated figure is entirely dependent upon the duration of the follow-up for reported case studies.

^3^Gurd et al. (2001) (case 2) report that “Neuropsychological examination showed verbal and performance IQs, short- and long-term memory, naming, reading and spelling skills which were within normal limits” (p. 715). However, for IQ measures and evaluation of mnestic functions, it is not clear which tests were presented.

^4^Madlen, Davies, “The woman with Foreign Accent Syndrome: Mother goes to bed with broad Staffordshire accent and wakes up sounding POLISH,” *MailOnline*, October 2^*nd*^ 2014, accessed on March 23^rd^, 2015, http://www.dailymail.co.uk/health/article-2778297/The-woman-Foreign-Accent-Syndrome-Mother-goes-bed-broad-Staffordshire-accent-wakes-sounding-POLISH.html “Embarrasing bodies, Conditions: Foreign Accent Syndrome,” *channel4embarrassingillnesses.com*, accessed on February 2^nd^, 2015; http://www.channel4embarrassingillnesses.com/conditions/foreign-accent-syndrome/Thomas, Emily, ‘Sarah Colwill Speaks Out About Foreign Accent Syndrome In BBC Documentary “The Woman Who Woke Up Chinese”’, *Huffingtonpost.com*, April 4^th^, 2013; accessed on 23^rd^ March, 2015; http://www.huffingtonpost.com/2013/09/04/sarah-colwill-_n_3869077.html

^5^no examples were provided.
